# Unilateral Pseudo–Double Side-by-Side Lined Macular Neovascularizations in Pathologic Myopia

**DOI:** 10.7759/cureus.101837

**Published:** 2026-01-19

**Authors:** Eda Hümaz, Seher Köksaldı, Mustafa Kayabaşı, Ali Osman Saatci

**Affiliations:** 1 Department of Ophthalmology, Dokuz Eylül University, İzmır, TUR; 2 Department of Ophthalmology, Ağrı İbrahim Çeçen University, Ağrı, TUR; 3 Department Ophthalmology, Muş State Hospital, Muş, TUR; 4 Department of Ophthalmology, Dokuz Eylül University, İzmir, TUR

**Keywords:** choroidal neovascular membrane, myopic macular neovascularization, optical coherence tomography, optical coherence tomography angiography, pathologic myopia

## Abstract

Pathologic myopia may be complicated by macular neovascularization (MNV) with atypical morphological configurations that can be challenging to characterize without multimodal imaging. A 50-year-old woman with pathologic myopia was examined due to a two-week history of newly developed left visual deterioration. Upon ophthalmological examination, her best-corrected Snellen visual acuity was hand motion in the right eye and 6/15 in the left eye. Slit-lamp examination revealed +4 nuclear sclerosis in the right eye, whereas the left eye appeared normal. Axial length measured by A-mode ultrasonography was 30.55 in the right eye and 28.04 in the left eye. The right fundus could be visualized only with indirect ophthalmoscopy, and there was severe patchy atrophy. There were two hyperpigmented side-by-side lined lesions indicating the presence of two macular neovascularizations at the left macula. Subfoveal spectral-domain optical coherence tomographic section depicted two hyperreflective materials located side by side at the outer retina, accompanied by mild intraretinal fluid. Optical coherence tomography angiography (OCTA) confirmed the presence of the pseudo-double MNV with an anatomical connection. The only good newly symptomatic left eye was treated with two intravitreal ranibizumab injections one month apart. The present case highlights that MNV associated with pathologic myopia may present with complex, pseudo-double configurations that can be accurately identified only through multimodal imaging. OCTA plays a crucial role in delineating the anatomical continuity and morphological characteristics of such lesions, thereby contributing to accurate diagnosis, treatment planning, and prognostic assessment.

## Introduction

A spherical refractive error exceeding −6.0 diopters and/or an axial length of at least 26.00 mm, coupled with characteristic degenerative changes in the sclera, choroid, and retinal pigment epithelium (RPE), defines pathologic myopia, a condition often associated with substantial visual loss during the course [1]. Pathologic myopia is fraught with macular complications such as posterior staphyloma, myopic maculopathy, and myopic macular neovascularization (MNV), with myopic MNV being among the most prevalent and vision-threatening sequelae of the condition [2,3].

MNV refers to the proliferation of abnormal blood vessels arising from the choroid and extending into or beneath the retina, where they may result in fluid leakage, hemorrhage, and subsequent fibrotic scarring, ultimately leading to central vision loss [1]. Traditionally, myopic MNV has been diagnosed using fluorescein angiography, indocyanine green angiography, and optical coherence tomography (OCT). Recently, optical coherence tomography angiography (OCTA) has emerged as a noninvasive alternative that does not require dye injection and enables en-face imaging with its segmentation capability for the detailed observation of MNV morphology and dimensions [4,5].

Typically, myopic MNV lesions are generally small, well-circumscribed, type 2 (classic) neovascular membranes located in the subfoveal or juxtafoveal region and typically present as a single focal lesion on both clinical examination and imaging [6]. However, neovascular complexes may occasionally exhibit an irregular or segmented growth pattern, appearing as separate lesions on fundus examination or structural OCT, despite originating from a single neovascular process [7,8]. These atypical presentations may cause diagnostic challenges.

In this brief report, we aim to present the multimodal findings of unilateral, apparently double but anatomically interconnected side-by-side MNVs, which we have named pseudo-double MNVs to indicate that the double appearance represents a morphological illusion rather than two independent neovascular entities. This case highlights the importance of OCTA in revealing the true vascular continuity underlying this unusual manifestation of pathologic myopia.

## Case presentation

**Figure 1:**
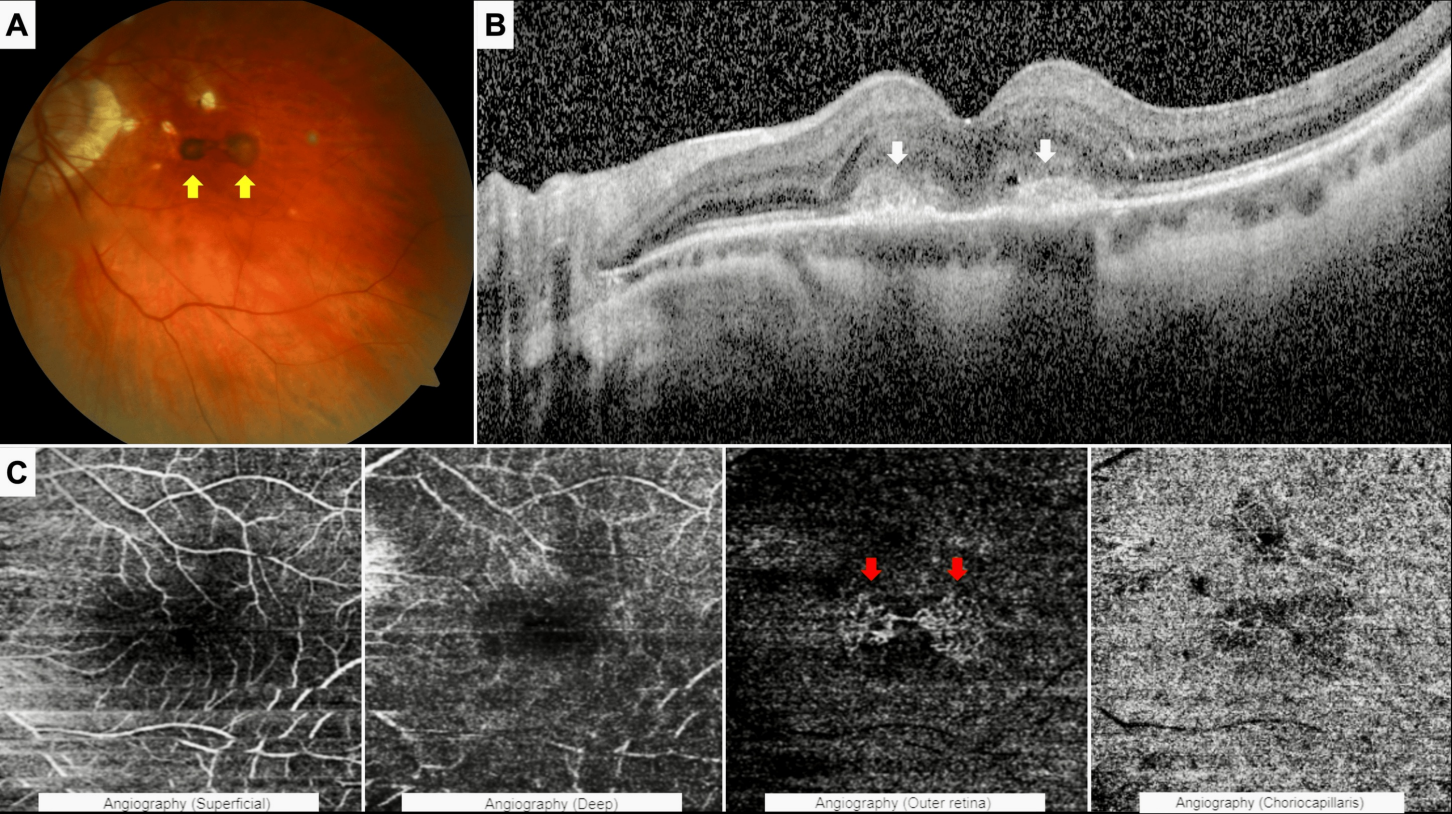
Left eye at the first presentation (A) Color fundus photograph showing two separate-looking hyperpigmented chorioretinal lesions (yellow arrows). (B) Spectral-domain optical coherence tomography images corresponding to the lesions, revealing the hyper-reflective material (white arrows), mild intraretinal fluid, and intraretinal cyst at the level of the outer retina. (C) Optical coherence tomography angiography 6 × 6 scans demonstrating different segmentation slabs: superficial capillary plexus (internal limiting membrane (ILM) + 2.6 µm to inner plexiform layer/inner nuclear layer (IPL/INL) + 15.6 µm), deep capillary plexus (IPL/INL + 15.6 µm to IPL/INL + 70.2 µm), outer retinal slab (IPL/INL + 70.2 µm to Bruch’s membrane (BM) + 0.0 µm), and choriocapillaris slab (BM + 0.0 µm to BM + 10.4 µm). The outer retinal slab displays a lacy vascular pattern consistent with a pseudo-double macular neovascularization, demonstrating anatomical continuity between the two apparent lesions (red arrows).

A 50-year-old woman with pathologic myopia was examined due to a two-week history of newly developed left visual deterioration. On examination, her best-corrected Snellen visual acuity was hand motion in the right eye and 6/15 in the left eye with a spectacle correction of −8.00 D −4.50 D × 100°. No autorefractive measurement could be obtained in the right eye. The intraocular pressure was 12 mmHg in both eyes. Biomicroscopic examination revealed +4 nuclear sclerosis in the right eye, whereas the left anterior segment appeared normal. Axial length measured by A-mode ultrasonography was 30.55 mm in the right eye and 28.04 mm in the left. Indirect ophthalmoscopy of the right eye revealed a category 3 myopic maculopathy, faintly, according to the meta-analysis for pathologic myopia [1]. There was a category 3 myopic maculopathy and two hyperpigmented grayish side-by-side macular lesions that appeared clinically as separate neovascular foci at the left of the macula, consistent with myopic MNVs (Figure 1A). Subfoveal, spectral-domain optical coherence tomographic (Heidelberg Spectralis, Heidelberg Engineering, Heidelberg, Germany) section depicted the presence of two hyperreflective materials located side by side at the outer retina, accompanied by mild intraretinal fluid (Figure 1B). While these findings suggested two distinct lesions on structural imaging, 6x6 mm macular OCTA images (Triton, Topcon Inc., Oakland, New Jersey, United States of America) confirmed the presence of a single anatomically interconnected neovascular complex with a pseudo-double configuration in the left eye (Figure 1C). She denied the right cataract surgery at first and demanded treatment for her good left eye. Thus, two intravitreal ranibizumab (0.5 mg) (Lucentis®, Novartis Pharma GmbH, Nürnberg, Germany) injections were administered one month apart.

**Figure 2:**
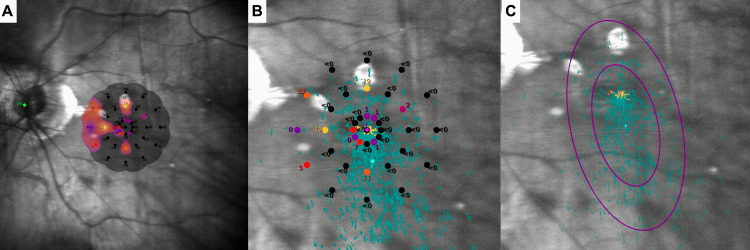
Macular integrity assessment microperimetry at the patient's last visit. Interpolated sensitivity map over full scanning laser ophthalmoscope image (A). Sensitivity values (decibel) and preferred retinal locus over zoomed scanning laser ophthalmoscope image (B). Fixation plot (turquoise dots) over zoomed scanning laser ophthalmoscope image, bivariate contour ellipse area 63 (small purple circle), and bivariate contour ellipse area 95 (large purple circle) (C).

Two months after the second injection left visual acuity was again 6/15, but she was subjectively feeling well. While no notable changes were observed in the left fundus, OCT revealed the resolution of the already mild intraretinal fluid. Macular Integrity Assessment (MAIA, CenterVue®, Padua, Italy) microperimetry was performed using the 4-2 strategy mode during the recent visit. The left macular integrity value was measured at 100 (abnormal), while the average threshold value was 1.6 decibels, indicating significant macular dysfunction. P1 value was 10%, and P2 value was 38%, indicating unstable fixation. The bivariate contour ellipse area (BCEA) measurements further illustrate this instability, 63% BCEA was 31.9°², 95% BCEA was 95.5°² (Figure 2).

## Discussion

Myopic MNV represents a serious vision-threatening complication and a common cause of central vision loss in pathologic myopia [9]. Its prevalence ranges between 5% and 11%, and approximately 15% of cases involve both eyes [3,10]. The condition is characterized by the occurrence of abnormal neovascularization beneath the RPE or within the retina [11].

In pathologic myopia, choroidal thinning is due to focal loss of the choriocapillaris, reduced number of large choroidal vessels, and loss of choroidal stroma [6]. It has been proposed that choroidal thinning may cause some hypoxic retinal changes, leading to vascular endothelial growth factor secretion and subsequent formation of MNV [6,8]. In addition, mechanical disruption of the Bruch membrane and the RPE adjacent to areas of patchy chorioretinal atrophy is likely to contribute to the MNV formation in pathologic myopia. Thus, mechanical structural lesions, hemodynamic alterations, hypoxic retinal changes, genetic risk factors, and systemic predisposing factors, either acting in combination or sequentially, may contribute to the development of MNV in pathologic myopia [6]. 

As seen in this case, myopic MNVs typically manifest as small, type 2 classic lesions near or under the fovea, leading to an initial rapid decline in vision, with or without associated metamorphopsia and central scotoma. Additionally, there is a predominance of females [12]. OCTA offers a groundbreaking, non-invasive, dye-free approach to directly and precisely visualize the morphology of MNV. It demonstrates a sensitivity of 90% to 94% in detecting MNV, with a specificity of 93.75% in patients with myopia [13]. On OCTA, myopic MNV appears as an abnormal network of interlacing or tangled vessels at the level of the outer retina and choriocapillaris [14].

Double neovascularization in the same eye was reported by Altinisik et al. [7] in a patient with pachychoroid neovasculopathy; one was an exudative lesion, and the second was quiescent. Similarly, Oliveira et al. [15] described two well-demarcated MNVs unilaterally in a patient with ocular tuberculosis. Yasukura et al. [16] reported a case with *Staphylococcus aureus* infective endocarditis where there was a single MNV in the right eye and three multifocal MNVs in the left eye. Likewise, Modjtahedi et al. [17] described a patient with metastatic melanoma receiving ipilimumab who developed bilateral peripapillary MNV and subfoveal MNV only in the left eye. In addition, Fung et al. [18] documented the presence of multiple MNV foci in the same eye of a patient with autosomal dominant familial exudative vitreoretinopathy. From a clinical perspective, pseudo-double or multiple MNV configurations may indicate a more complex neovascular process, characterized by a broader area of chorioretinal involvement and potentially heterogeneous activity within the same macular region. This anatomical complexity may influence treatment response, as different components of the lesion could regress at variable rates following anti-vascular endothelial growth factor therapy. Moreover, extensive neovascular tissue adjacent to areas of patchy atrophy may theoretically increase the risk of subsequent macular atrophy or fibrotic scarring, thereby limiting long-term visual recovery despite apparent anatomical improvement. 

From a functional standpoint, microperimetry demonstrated markedly reduced macular sensitivity and unstable fixation, as reflected by low mean threshold values, decreased P1 and P2 percentages, and enlarged BCEA measurements. These findings indicate substantial macular dysfunction despite relatively preserved best-corrected visual acuity, underscoring the limitation of visual acuity alone in accurately reflecting functional impairment in myopic MNV. Although a direct spatial correlation between areas of sensitivity loss and the pseudo-double neovascular complex could not be established, the pronounced functional deficit suggests more widespread macular involvement, potentially related to the anatomical complexity of the lesion.

Several limitations should be acknowledged when interpreting these observations. Follow-up OCT and OCTA images were not available for this patient. In addition, quantitative vascular parameters such as vessel density or flow-related metrics could not be evaluated, as the OCTA software utilized in this case does not provide such metrics for outer retinal layers. Accordingly, the primary aim of this report was not to perform a quantitative or longitudinal analysis, but rather to emphasize the distinctive pseudo-double morphological configuration of MNV in a patient with pathologic myopia. Notably, although exudation resolved after treatment, visual acuity improvement was limited, suggesting that pseudo-double MNVs may be associated with functional impairment disproportionate to their exudative activity. Identification of such configurations may carry prognostic significance and support the need for closer follow-up in patients with pathologic myopia.

## Conclusions

The present case highlights that MNV associated with pathologic myopia may present with atypical, pseudo-double side-by-side configurations that are not always readily apparent on conventional clinical examination alone. Multimodal imaging, particularly OCTA, enables precise visualization of lesion morphology and anatomical continuity, thereby facilitating accurate diagnosis and informed management. Although the functional impact observed in this case suggests that such anatomically complex configurations may be associated with disproportionate macular dysfunction, further studies are needed to clarify their prognostic significance. Awareness of such complex presentations may assist clinicians in anticipating functional outcomes and underscores the importance of meticulous, imaging-based evaluation in patients with pathologic myopia.
